# Emerging Tickborne Bacteria in Cattle from Colombia

**DOI:** 10.3201/eid2810.220657

**Published:** 2022-10

**Authors:** Alejandro Ramírez-Hernández, Esteban Arroyave, Álvaro A. Faccini-Martínez, Heidy C. Martínez-Diaz, Paola Betancourt-Ruiz, Luz-Adriana Olaya-M, Elkin G. Forero-Becerra, Marylin Hidalgo, Lucas S. Blanton, David H. Walker

**Affiliations:** Universidad de La Salle, Bogotá, Colombia (A. Ramírez-Hernández);; University of Texas Medical Branch, Galveston, Texas, USA (A. Ramírez-Hernández, E. Arroyave, Á.A. Faccini-Martínez, L.S. Blanton, D.H. Walker);; Fundación Universitaria de Ciencias de la Salud, Bogotá (Á.A. Faccini-Martínez);; Servicios y Asesorías en Infectología Sai S.A.S., Bogotá (Á.A. Faccini-Martínez);; Pontificia Universidad Javeriana, Bogotá (H.C. Martínez-Diaz, P. Betancourt-Ruiz, E.G. Forero-Becerra, M. Hidalgo);; Universidad Libre, Cali, Colombia (L.-A. Olaya-M)

**Keywords:** bacteria, parasites, vector-borne infections, zoonoses, tickborne diseases, cattle, *Borrelia theileri*, *Ehrlichia minasensis*, anaplasmosis, tandem repeat protein 36, Trp36, Colombia

## Abstract

*Ehrlichia minasensis* is a new pathogenic bacterial species that infects cattle, and *Borrelia theileri* causes bovine borreliosis. We detected *E. minasensis* and *B*. *theileri* DNA in cattle from southwestern Colombia by using PCR. *E. minasensis* and *B*. *theileri* should be considered potential etiologies of febrile syndrome in cattle from Colombia.

*Ehrlichia* spp. are tickborne obligate intracellular bacteria and comprise different pathogenic species that affect both veterinary and public health (*1*). *Ehrlichia minasensis* was first detected in cattle and deer in Canada and later in cattle and *Rhipicephalus microplus* ticks from Brazil (*2*–*4*). Infected cattle manifest signs that include fever, lethargy, depression, and anorexia (*3*,*4*). *Borrelia theileri* belongs to the relapsing fever group of borreliae and causes bovine borreliosis, which is a mild febrile disease associated with lethargy, hemoglobinuria, and anemia (*5*). This spirochete is transmitted by *Rhipicephalus* (formerly *Boophilus*) sp. ticks and has been documented in Africa, Europe, Oceania, and South America (*5*,*6*). To our knowledge, *E*. *minasensis* or *B. theileri* infections have not been reported in cattle from Colombia. 

During September and October 2017, we collected blood samples from 30 bovids with tick parasitism in El Tambo and Santander de Quilichao municipalities, Cauca department, Colombia (Appendix Figure). We extracted DNA from blood using the QIAGEN DNeasy Blood & Tissue Kit (https://www.qiagen.com), according to the manufacturer’s instructions. We verified DNA quality using PCR amplification of the vertebrate cytochrome B gene *CYTB*. We subsequently performed PCR to detect *dsb* and *trp36* genes for *Ehrlichia* spp.; *flaB* and 16S rRNA genes for *Borrelia* spp.; and *rpoB*, *msp4*, and *msp1a* genes for *Anaplasma* spp. (Appendix Table). DNA samples that produced strong PCR bands underwent sequencing on an Applied Biosystems 3130/3130xl Genetic Analyzer (Thermo Fisher Scientific, https://www.thermofisher.com). The *msp1a* PCR products were poor quality and did not undergo sequencing. We aligned sequences using GeneStudio (GeneStudio, Inc., https://genestudio-pro.software.informer.com) and performed multiple sequence alignments using the EMBL-European Bioinformatics Institute tools MUSCLE (for *Ehrlichia* spp.) and ClustalW (for *Borrelia* and *Anaplasma* spp.) (https://www.clustal.org). Phylogenetic analyses were performed with MEGA X software (https://www.megasoftware.net). We generated phylogenetic trees for *dsb*, *flaB*, and 16S rRNA genes and Trp36 protein using the maximum-likelihood estimation method. All procedures were approved by the Pontificia Universidad Javeriana Ethics Committee in Colombia.

We detected *CYTB* in all samples. We detected the *dsb* gene in 10% (3/30) and *trp36* in 20% (6/30) of samples. The *flaB* gene was detected in 13.3% (4/30), and the 16S rRNA gene was detected in 10% (3/30) of samples. The *rpoB* gene was amplified in 90% (27/30), *msp4* was amplified in 83.3% (25/30), and *msp1a* was amplified in 83.3% (25/30) of samples. We performed phylogenetic analyses of 3 sequences for *dsb* (GenBank accession nos. ON209405–7), 6 sequences for Trp36 protein (inferred from GenBank accession nos. OL513405–10), 3 sequences for the 16S rRNA gene (GenBank accession nos. ON112216–8), 4 sequences for *flaB* (GenBank accession nos. ON135431–4); 6 sequences for *rpoB* (GenBank accession nos. ON209412–7), and 4 sequences for *msp4* (GenBank accession nos. ON209408–11). 

Phylogenetic analyses showed that our *dsb* gene sequences clustered with *E. minasensis* *dsb* sequences from Brazil, Australia, and Colombia (Figure, panel A). The greater genetic diversity of Trp36 protein compared with *dsb* provided more detailed characterization of *Ehrlichia* sp. genotypes. Our Trp36 sequences clustered with 3 sequences from Brazil and a recently described *E. minasensis* strain from China isolated from *Haemaphysalis hystricis* ticks (Figure, panel B). The *flaB* and 16S rRNA genes clustered with *B*. *theileri* sequences (Figure, panels C, D). Bootstrap values were 86% for *flaB* and 72% for the 16S rRNA gene. The *flaB* sequences grouped with sequences from Argentina, Republic of the Congo, Egypt, and Israel (Figure, panel C). The 16S rRNA gene sequences grouped independently but close to sequences from Egypt and the United States (Figure, panel D). We confirmed *A*. *marginale* using identity analysis (100% identical to GenBank sequences; accession nos. CP023731, CP006846, CP001079, CP000030, and AF428086) of *rpoB* and *msp4* genes. Co-infection with *E. minasensis* and *A. marginale* was confirmed in 6 animals from El Tambo. Co-infection with *B*. *theileri* and *A*. *marginale* was documented in 1 animal from El Tambo and 1 animal from Santander de Quilichao.

**Figure:**
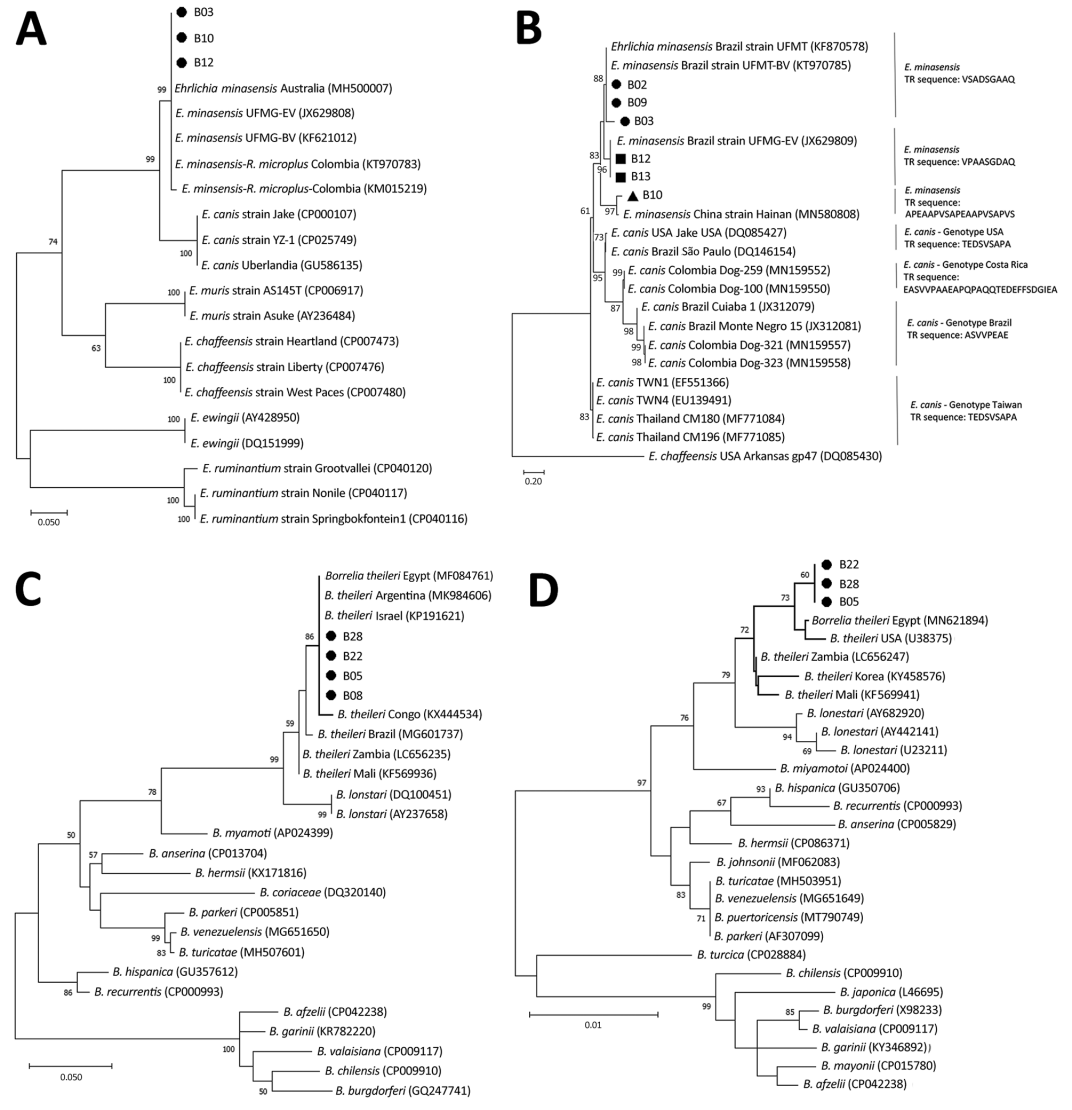
Phylogenetic analysis of emerging tickborne bacteria in cattle from Colombia. Phylogenetic trees are shown for *dsb* genes (A), Trp36 proteins (amino acid sequences) (B), *flaB* genes (C), and 16S rRNA genes (D). We performed PCR on blood samples from cattle in El Tambo and Santander de Quilichao, Cauca department, Colombia to detect *dsb* and *trp36* genes for *Ehrlichia* sp.; *flaB* and 16S rRNA genes for *Borrelia* sp.; and *rpoB*, *msp4*, and *msp1a* genes for *Anaplasma* sp. We generated phylogenetic trees using the maximum-likelihood method and Tamura 3-parameter model with a gamma distribution parameter of 0.28 to compare evolutionary relationships between our sequences and publicly available sequences from Genbank (accession numbers indicated on trees). We applied bootstrap tests using 1,000 replicates; bootstrap values are shown at key nodes. The ●, ■, and ▲ symbols represent the differential clustering of sequences obtained in this study. Scale bars indicate nucleotide substitutions per site.

These results showed the simultaneous circulation of *E. minasensis*, *B*. *theileri*, and *A. marginale* in bovids from Cauca department, Colombia. In Latin America, *E. minasensis* has been identified in Brazil (*7*) and Colombia (found in *R*. *microplus* ticks) (*8*), and *B*. *theileri* has been found in Argentina (*6*), Mexico (*9*), and Brazil (*10*). The *R. microplus* tick is likely the main vector for both pathogens in these regions and has been confirmed by molecular detection of *E*. *minasensis* in tick specimens collected from the same animals (H.C. Martínez-Diaz, unpub. data) and various reports in Latin America for *B*. *theileri* (*9*,*10*). Despite the lack of clinical signs in these animals, tickborne infections caused by these pathogenic bacteria often occur as subclinical infections or with intermittent clinical manifestations. *E. minasensis* and *B*. *theileri* infections, either separately or as co-infections, may be more frequent than previously recognized and should be considered potential etiologies of febrile syndrome in cattle from this and other regions of Colombia.

Appendix Additional information for emerging tickborne bacteria in cattle from Colombia.
