# Supplementary material for: Emerging Tickborne Bacteria in Cattle from Colombia
Source: Emerg Infect Dis. 2022 Oct;28(10):2109–11. doi: 10.3201/eid2810.220657 (PMC9514337; doi:10.3201/eid2810.220657)
Supplement: Appendix — Additional information for emerging tickborne bacteria in cattle from Colombia. [file 22-0657-Techapp-s1.pdf]

Article DOI: https://doi.org/10.3201/eid2810.220657

# Emerging Tickborne Bacteria in Cattle from Colombia

## Appendix

**Appendix Table.** List of PCR primers used to identify emerging tickborne bacteria in cattle from Colombia

| Target gene | Primer sets | Sequence (5′–3′) | Annealing temperature (°C) | References |
|---|---|---|---|---|
| *CYTB** | Cyt bF | CCCCTCAGAATGATATTTGTCCTCA | 60 | (*1*) |
| | Cyt bR | CCATCCAACATCTCAGCATGATGAAA | | |
| *dsb* (heminested) | Dsb-330 (first reaction) | GATGATGTCTGAAGATATGAAACAAAT | 52 | (*2*,*3*) |
| | Dsb-380 (second reaction) | ATTTTTAGRGATTTTCCAATACTTGG | | |
| | Dsb-720 (both reactions) | CTATTTTACTTCTTAAAGTTGATAWATC | | |
| *trp36* (heminested) | TRP36-F2 (first reaction) | TTTAAAACAAAATTAACACACTA | 45 | (*4*,*5*) |
| | TRP36-DF (second reaction) | CACACTAAAATGTATAATAAAGC | | |
| | TRP36-R1 (both reactions) | AAGATTAACTTAATACTCAATATTACT | | |
| *flaB* (nested) | FlaLL (first reaction) | ACATATTCAGATGCAGACAGAGGT | 55 | (*6*) |
| | FlaRL (first reaction) | GCAATCATAGCCATTGCAGATTGT | | |
| | FlaLS (second reaction) | AACAGCTGAAGAGCTTGGAATG | 55 | |
| | FlaRS (second reaction) | CTTTGATCACTTATCATTCTAATAGC | | |
| 16S rRNA (nested) | B1 (first reaction) | CAGTGCGTCTTAAGCATGC | 56 | (*7*) |
| | B8 (first reaction) | CCTTAAATACCTTCCTCCC | | |
| | B3 (second reaction) | GCAGCTAAGAATCTTCCGCAATGG | 59 | |
| | B6 (second reaction) | CAACCATGCAGCACCTGTATAT | | |
| *rpoB* | Ana-rpoBF | GCTGTTCCTAGGCTYTCTTACGCGA | 55 | (*8*) |
| | Ana-rpoBR | AATCRAGCCAVGAGCCCCTRTAWGG | | |
| *msp4* | MSP45 | GGGAGCTCCTATGAATTACAGAGAATTGTTTAC | 60 | (*9*) |
| | MSP43 | CCGGATCCTTAGCTGAACAGGAATCTTGC | | |
| *msp1a* | MSP1aP | GCATTACAACGCAACGCTTGAG | 68 | (*9*) |
| | MSP1a3 | GCTTTACGCCGCCGCCTGCGCC | | |

*Primer set was developed to detect cytochrome B genes in multiple vertebrate species, including cattle. Primer names were not provided in the original publication.

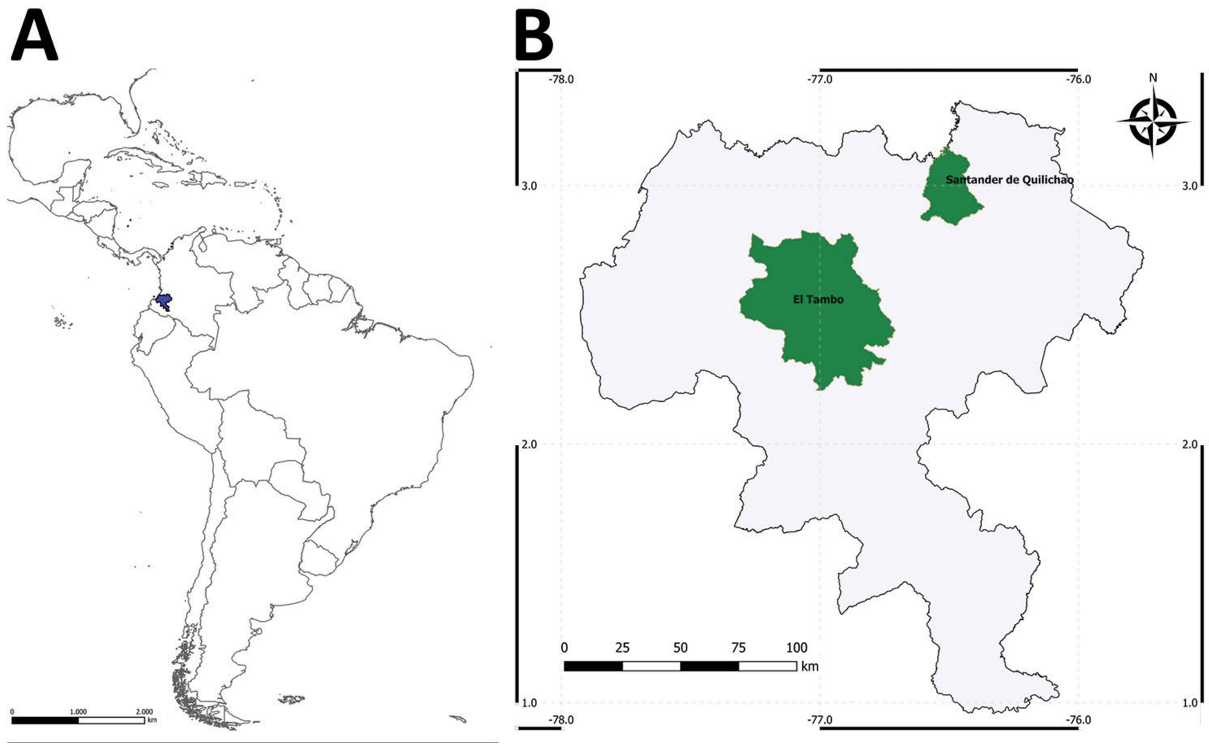


**Appendix Figure**. Maps showing locations where emerging tickborne bacteria *Ehrlichia minasensis* and *Borrelia theileri* were found in cattle from El Tambo and Santander de Quilichao municipalities in Cauca department, Colombia. We collected blood samples from cattle and performed PCR analysis to detect different genes specific for *Ehrlichia minasensis*, *Borrelia theileri*, and *Anaplasma* sp. A) Blue area indicates Cauca department within Colombia. B) Green areas indicate El Tambo and Santander de Quilichao municipalities within Cauca department, Colombia.

## References

1. Boakye DA, Tang J, Truc P, Merriweather A, Unnasch TR. Identification of bloodmeals in haematophagous *Diptera* by cytochrome B heteroduplex analysis. Med Vet Entomol. 1999;13:282–7. PubMed https://doi.org/10.1046/j.1365-2915.1999.00193.x

2. Doyle CK, Labruna MB, Breitschwerdt EB, Tang YW, Corstvet RE, Hegarty BC, et al. Detection of medically important *Ehrlichia* by quantitative multicolor TaqMan real-time polymerase chain reaction of the *dsb* gene. J Mol Diagn. 2005;7:504–10. PubMed https://doi.org/10.1016/S1525-1578(10)60581-8

3. Almeida AP, Souza TD, Marcili A, Labruna MB. Novel *Ehrlichia* and *Hepatozoon* agents infecting the crab-eating fox (*Cerdocyon thous*) in southeastern Brazil. J Med Entomol. 2013;50:640–6. PubMed https://doi.org/10.1603/ME12272

4. Aguiar DM, Zhang X, Melo AL, Pacheco TA, Meneses AM, Zanutto MS, et al. Genetic diversity of *Ehrlichia canis* in Brazil. Vet Microbiol. 2013;164:315–21. PubMed https://doi.org/10.1016/j.vetmic.2013.02.015

5. Aguiar DM, Ziliani TF, Zhang X, Melo AL, Braga IA, Witter R, et al. A novel *Ehrlichia* genotype strain distinguished by the *TRP36* gene naturally infects cattle in Brazil and causes clinical manifestations associated with ehrlichiosis. Ticks Tick Borne Dis. 2014;5:537–44. PubMed https://doi.org/10.1016/j.ttbdis.2014.03.010

6. Moore VA 4th, Varela AS, Yabsley MJ, Davidson WR, Little SE. Detection of *Borrelia lonestari*, putative agent of southern tick-associated rash illness, in white-tailed deer (*Odocoileus virginianus*) from the southeastern United States. J Clin Microbiol. 2003;41:424–7. PubMed https://doi.org/10.1128/JCM.41.1.424-427.2003

7. Takhampunya R, Thaloengsok S, Tippayachai B, Promsathaporn S, Leepitakrat S, Gross K, et al. Retrospective survey of *Borrelia* spp. from rodents and ticks in Thailand. J Med Entomol. 2021;58:1331–44. PubMed https://doi.org/10.1093/jme/tjaa279

8. Dahmani M, Davoust B, Rousseau F, Raoult D, Fenollar F, Mediannikov O. Natural Anaplasmataceae infection in *Rhipicephalus bursa* ticks collected from sheep in the French Basque Country. Ticks Tick Borne Dis. 2017;8:18–24. PubMed https://doi.org/10.1016/j.ttbdis.2016.09.009

9. de la Fuente J, Van Den Bussche RA, Kocan KM. Molecular phylogeny and biogeography of North American isolates of *Anaplasma marginale* (Rickettsiaceae: Ehrlichieae). Vet Parasitol. 2001;97:65–76. PubMed https://doi.org/10.1016/S0304-4017(01)00378-8
